# The Effects of Reflexology on Fatigue Severity of Patients with Cancer

**DOI:** 10.31557/APJCP.2019.20.2.391

**Published:** 2019

**Authors:** Hassan Nourmohammadi, Minoo Motaghi, Milad Borji, Asma Tarjoman, Behrouz Soltany

**Affiliations:** 1 *Medical Oncologist and Hematologist, Department of Internal Medicine, Ilam University of Medical Science, Ilam, *; 2 *Nursing Department, Faculty of nursing and Midwifery, Isfahan (Khorasgan) Branch, Islamic Azad University, Isfahan, *; 3 *Department of Nursing, Faculty of Nursing and Midwifery, *; 4 *Students Research Committee, Kermanshah University of Medical Science, Kermanshah, Iran. *

**Keywords:** Fatigue severity, reflexology, breast cancer

## Abstract

**Introduction:**

Breast cancer is a major threat to women’s health and a common factor that can reduce their life expectancy. Complementary medicine such as reflexology is known to reduce fatigue severity in cancer patients. The present study aimed to cultivate the effect of reflexology on fatigue severity of patients with breast cancer.

**Methods:**

The present pre-post clinical trial recruited 57 patients with breast cancer and involved an experimental and a control group. All patients were livening in Ilam, Iran. Patients were randomly assigned to two groups of experimental (N=27) and control (N=30). The experimental group received reflexology for 4 sessions. Data were collected using Fatigue severity scale (FSS) and demographic information questionnaire. FSS was completed by the patients twice; before the intervention and 2 months after the intervention. Data were analyzed using SPSS and running t-test and ANOVA.

**Results:**

Results showed no significant difference in fatigue severity between experimental (45.44±5.30) and control (43.66±7.68) groups prior to the intervention (p>0.05). However, after conducting the intervention, a significant difference in fatigue severity was seen between the experimental (20.66±4.54) and control (40.36±9.58) groups (p=0.000).

**Conclusion:**

The present study showed that reflexology decrease fatigue severity in patients with breast cancer and community health nursing can to use of these complementary medicine to increase patients health conditions.

## Introduction

Cancer is an important healthcare issue worldwide because it is the third leading cause of death and the second most common chronic disease (Rossi et al., 2017; Shahrbabaki et al., 2012; Khoshnood et al., 2018; Safizadeh et al., 2018). In fact, estimated deaths and complications caused by cancer are on rise (Organization, 2015; Khoshnood et al., 2018; Pourdeghatkar et al., 2017). Breast cancer is a major threat in particular to women’s health and a common risk factor that can decrease life expectancy among sufferers (Parkin et al., 1999). In Iran, many women deaths was reported due to breast cancer (Reyhani et al., 2012). One common symptom among patients with breast cancer is fatigue (Reinertsen et al., 2010), 70% of which is caused by chemotherapy and radiotherapy (Rad et al., 2016).

Previous studies have indicated that fatigue decreases the quality of life in patients with cancer that is associated with lower survival rate and higher mortality rate. In addition, fatigue may affect patient’s physical activity, increase his/her sense of loneliness and isolation, and interfere with daily living activities (Barsevick et al., 2008; Rad et al., 2016). In recent years, non-drug therapies have been used in combination with drug therapy to decrease fatigue in patients with cancer (Mustian et al., 2011). Patients themselves can try complementary medicine, which increases their independence. Furthermore, in comparison with drug therapy, complementary medicine has lower rates of negative side effects and consequences (Zargarzadeh and Memarian, 2013). Consequently, complementary medicine is becoming more popular globally. In most regions of Africa, Asia, and Latin America, it is among the most important methods of care and treatment available to patients (Zargarzadeh and Memarian, 2013). 

Common interventions suggested to reduce fatigue are yoga (Vadiraja et al., 2017; Sadja and Mills, 2013), cognitive-behavioral therapy (Sandler et al., 2017, Montgomery et al., 2014), acupressure (Bastani et al., 2015), exercise (Shariati et al., 2010), and reflexology (Jin and Kim, 2005; Özdelikara and Tan, 2017). Reflexology is a treatment in which practitioners apply pressure to the feet or hands using their fingers. It is used to treat various symptoms, such as pain, anxiety, and fear (Valiani et al., 2010; Özdemir et al., 2013; Hudson et al., 2015; Hughes et al., 2009; Bagheri-Nesami et al., 2014). This therapy increases patient comfort by inducing physiological changes. Specifically, the pressure on the reflexive areas stimulates hundreds of nerve endings in the soles, causing the release of endorphins, and consequently prevents pain transfer, causes comfort and anesthesia, decreases tension, and increases tranquility (Pitman and MacKenzie, 2002). Given high prevalence of breast cancer in Iran and the importance of complementary medicine in enhancing patients’ health, this study aimed to investigate the effect of reflexology on fatigue severity in patients with breast cancer. 

## Materials and Methods

The present investigation was a pre-post, double-blind study, involving experimental and control groups. The study was conducted on patients with breast cancer living in Ilam, Iran, during at least previous 8 month. A total of 60 patients with a definitive breast cancer diagnosis were randomly recruited for the study. All the patients were in the first stage of breast cancer and received chemotherapy. The inclusion criteria were as follows: (1) definitive breast cancer diagnosis, (2) absence of metastasis according to oncologist’ diagnosis, (3) absence of cardiovascular disease, (4) absence of bone fractures, skin lesions, or disorders, (5) absence of mental disorders, (6) absence of diabetes mellitus, (7) having cancer for at least one year, having contact information, and (9) residing in Ilam, Iran .

The exclusion criteria were as follows: (1) non-cooperation of patients during the intervention, (2) absence for more than three sessions, (3) using other methods of complementary or alternative medicine, and (4) suffering from any crises affecting the patients’ fatigue severity. The study was initiated after receiving permission from the Research Ethics Committee. The selected Patients were divided randomly into two groups of experimental group (reflexology group) and control group. To prevent information transfer between two groups, the randomization was done based on the days of week. Specifically, at the beginning of every week, we randomly selected four days and allocated them to reflexology group. During the interventions, three patients from experimental group were excluded from the study and the study was continued with 27 patients in the experimental group and 30 patients in the control group.

For data collection, demographic information questionnaire and fatigue severity scale (FSS) were used. Demographic information questionnaire has 9 questions. Totally, the scores range between 0 and 63, and higher scores reveal higher severity of fatigued (Shahid et al., 2011). Experimental group received reflexology twice a week for 4 consecutive weeks while each session lasted for 20 minutes. To prepare patients’ legs before performing reflexology, the patients carried out movements such as bending the legs up and down, stretching the calves and ankles, turning the ankles inward and outward, and flexing the feet. General reflexology was performed using a worm-like movement of the thumb and index finger on reflexive areas. Specialized reflexology was then performed by pressing the major reflexive points of the soles (solar plexus, pituitary gland, spinal cord, vertebral column, pelvis, and limbs) with the thumb and index finger. The toes and other reflexive areas of the soles, as well as the back of the feet were also massaged. Pressing the thumb on the solar plexus signaled the end of the intervention (Soheili shahreza et al., 2014). Control group received routine care and the prescribed medication by the oncologist. The FSS was re-filled 2 months after the intervention.

Written informed consent was obtained from each patient and no costs were imposed upon patients for receiving reflexology. The study followed the Declaration of Helsinki and Belmont Report and research publication ethics were observed. Data were analyzed using SPSS (version 16) and running t-test and ANOVA .

## Results

There was no significant difference among the 2 study groups in terms of demographic information before conducting the intervention (p>0.05) (Table1).

**Table 1:** Demographic Information of Patients with Breast Cancer in the Two Study Groups

Variable	Group		p-value
	Reflexology	Contorol	
Age (Mean (SD))	47.85 (8.39)	50.86 (6.50)	P=0.13
Maritl status			P=0.89
Has spouse	24 (88.9)	27 (90)	
No spouse	3 (11.1)	3 (10)	
Education Level			P=0.32
Illiterate	6 (22.2)	9 (30)	
Diploma	16 (59.3)	17 (56.7)	
Collegiate	5 (18.5)	4 (13.3)	
Annual Icome			P=0.45
Weak	22 (81.5)	22 (73.3)	
medium	4 (14.8)	6 (20)	
Good	1 (3.7)	2 (6.7)	
Family support			P=0.56
Weak	2 (6.7)	4 (14.8)	
medium	19 (63.3)	10 (37)	
Good	9 (30)	13 (48.1)	
Believes in the impact Palliative interventions			P=0.13
Yes	18 (66.7)	14 (46.7)	
No	9 (33.3)	16 (53.3)	

**Table 2:** Comparison of Patients’ Fatigue Severity before and after the Intervention

Variable	Reflexology	Control	P , T
	Mean (SD)	Mean (SD)	
Fatigue severity			
Before	45.44±5.30	43.66±7.68	P=0.31 T= 1.05
After	20.66±4.54	40.36±9.58	P=0.000 T= -9.73
p-value	P=0.000	P=0.16	

As shown in Table 2, no significant difference in fatigue severity was observed between the experimental (45.44±5.30) and control (43.66±7.68) groups prior to conduct the intervention (p>0.05). However, after the intervention, a significant difference in fatigue severity was seen between the experimental (20.66±4.54) and control (40.36±9.58) groups (p=0.000).

## Discussion

The present study showed that reflexology decreased fatigue severity in patients with cancer, which is similar to findings reported by the previous studies, suggesting that reflexology can improve the general health of patients. For instance, Özdemir G et al., (2013) addressed fear, pain, and fatigue in patients undergoing hemodialysis and concluded that reflexology had a positive effect on their fatigue and could decrease its severity. Hughes et al., (2009) investigated pain in patients with multiple sclerosis and found that reflexology improved patients’ conditions and reduced their pain and fatigue. Furthermore, various studies indicated that reflexology improved the health of cancer patients. For example, Stephenson et al., (2007) revealed that reflexology decreased pain in patients with cancer, and Tsay et al., (2008) demonstrated that the therapy ameliorated both anxiety and pain in patients with cancer. 

In particular, Özdemir et al., (2013) conducted a study to determine the effect of reflexive sole massage on fatigue severity, pain, and muscle cramps in patients undergoing hemodialysis. The results suggested that reflexology decreased fatigue severity, muscle cramps, and pain in these patients. Yang et al., (2005) and Roshanravan et al., (2016) found that reflexology on the soles decreased fatigue, supporting the findings of the present study. Moreover, reflexology appears to cause physiological changes that increase comfort in patients (Jin and Kim, 2005). 

In conclusion, the present study showed that reflexology decreased fatigue severity in patients with breast cancer. Therefore, community health nursing staff are suggested to use this complementary medicine to enhance patients’ health conditions.
